# Reduced susceptibility of clinical strains of *Mycobacterium tuberculosis* to reactive nitrogen species promotes survival in activated macrophages

**DOI:** 10.1371/journal.pone.0181221

**Published:** 2017-07-13

**Authors:** Jonna Idh, Blanka Andersson, Maria Lerm, Johanna Raffetseder, Daniel Eklund, Hanna Woksepp, Jim Werngren, Mikael Mansjö, Tommy Sundqvist, Olle Stendahl, Thomas Schön

**Affiliations:** 1 Division of Microbiology and Molecular Medicine, Department of Clinical and Experimental Medicine, Linköping University, Linköping, Sweden; 2 Centre for Infectious Medicine, Karolinska Institute, Stockholm, Sweden; 3 Department of Optometry and Medicine, Linneaus University, Kalmar, Sweden; 4 Department of Infectious Diseases and Clinical Microbiology, Kalmar County Hospital, Kalmar, Sweden; 5 Department of Microbiology, Unit for Laboratory Surveillance of Bacterial Pathogens, Public Health Agency of Sweden, Solna, Sweden; Institut de Pharmacologie et de Biologie Structurale, FRANCE

## Abstract

**Background:**

Drugs such as isoniazid (INH) and pretomanid (PRT), used against *Mycobacterium tuberculosis* are active partly through generation of reactive nitrogen species (RNS). The aim of this study was to explore variability in intracellular susceptibility to nitric oxide (NO) in clinical strains of *M*. *tuberculosis*.

**Method:**

Luciferase-expressing clinical *M*. *tuberculosis* strains with or without INH resistance were exposed to RNS donors (DETA/NO and SIN-1) in broth cultures and bacterial survival was analysed by luminometry. NO-dependent intracellular killing in a selection of strains was assessed in interferon gamma/lipopolysaccharide-activated murine macrophages using the NO inhibitor L-NMMA.

**Results:**

When *M*. *tuberculosis* H37Rv was compared to six clinical isolates and CDC1551, three isolates with *inhA* mediated INH resistance showed significantly reduced NO-susceptibility in broth culture. All strains showed a variable but dose-dependent susceptibility to RNS donors. Two clinical isolates with increased susceptibility to NO exposure in broth compared to H37Rv were significantly inhibited by activated macrophages whereas there was no effect on growth inhibition when activated macrophages were infected by clinical strains with higher survival to NO exposure in broth. Furthermore, the most NO-tolerant clinical isolate showed increased resistance to PRT both in broth culture and the macrophage model compared to H37Rv in the absence of mutational resistance in genes associated to reduced susceptibility against PRT or NO.

**Conclusion:**

In a limited number of clinical *M*. *tuberculosis* isolates we found a significant difference in susceptibility to NO between clinical isolates, both in broth cultures and in macrophages. Our results indicate that mycobacterial susceptibility to cellular host defence mechanisms such as NO need to be taken into consideration when designing new therapeutic strategies.

## Introduction

It has been estimated that only a few percent of individuals infected with *Mycobacterium tuberculosis* actually develop active tuberculosis (TB) during their lifetime. This suggests that effective immune responses are protecting the host from developing disease [1]. Understanding such host mechanisms and their interactions with current antibiotics used against TB is becoming increasingly important from a therapeutic perspective, since multidrug-resistant (MDR) strains of *M*. *tuberculosis* are a growing health emergency [2].

Nitric oxide (NO) is produced by the inducible nitric oxide synthase (iNOS) in macrophages activated by pro-inflammatory cytokines [3]. In conjunction with the production of reactive oxygen species during inflammation, NO gives rise to other reactive nitrogen species (RNS) such as peroxynitrite (ONOO^-^), formed by NO and the superoxide anion (O_2_^-^) [3]. In human TB, the relative importance of macrophage-derived NO in host defence against TB is not clear, but it has previously been shown that patients with pulmonary TB have the capacity to produce NO locally in the lung [4, 5]. In mouse models, the importance of NO is well established, and mice lacking iNOS develop a more disseminated disease with reduced survival following *M*. *tuberculosis* infection [6].

It has recently been shown that in addition to the role of host-derived NO as a mediator of intracellular killing of *M*. *tuberculosis*, the activity of drugs such as isoniazid (INH) and pretomanid (PRT; PA-824) partly involves generation of NO [7–9]. Both PRT and INH need to be activated by mycobacterial factors [7–9]. INH requires activation by the mycobacterial catalase-peroxidase (Kat G), converting the drug to its active form along with generation of NO [10]. PRT is a bicyclic nitroimidazol whose anaerobic activity is mediated by the release of NO through conversion of the drug by a bacterial deazaflavin-dependent nitroreductase (Ddn) [7, 8]. Thus, variability in mycobacterial susceptibility against NO may influence the efficacy of important drugs such as PRT and INH.

The aim of this study was to explore NO susceptibility of clinical *M*. *tuberculosis* strains both inside macrophages and in broth cultures, and to explore how this is correlated to the antimicrobial activity of INH and PRT.

## Methods

### Culture and activation of macrophages

Murine macrophages (RAW 264.7; American Type Culture Collection) were cultured in DMEM (L-glutamine, pyruvate and glucose) supplemented with 10% heat-inactivated foetal bovine serum (FBS), 100 U/ml penicillin and 100 U/ml streptomycin (Gibco). When harvested, cells were counted and seeded in 96-well plates (Sarstedt) in medium free of antibiotics (25 000 cells/well). Cells were incubated with or without interferon gamma (IFN-γ, 2500 U/ml; Nordic Biosite) and lipopolysaccharide (LPS, 100 ng/ml; Sigma) for 5 hours before infection and then infected in the presence of 1250 U/ml IFN-γ and 50 ng/ml LPS. In order to investigate the effect of iNOS inhibition, cells were pre-incubated with or without 1mM of the arginine derivative L-NMMA (Sigma-Aldrich) for one hour before adding the IFN-γ and LPS.

### Bacteria

The virulent *M*. *tuberculosis* strain H37Rv (American Type Culture Collection, ATCC 27294) harbouring a pSMT1-plasmid encoding *Vibrio harveyi* luciferase was grown and prepared as previously described [11]. Clinical strains (Table 1) were transformed with the luciferase encoding pSMT1-plasmid as described by Garbe *et al*. [12]. In short, plasmid DNA was prepared from recombinant *E*. *coli* DH5α and used to electroporate the clinical strains. Successfully transformed strains were stored at −80°C in 25% glycerol. All strains were cultured in Middlebrook 7H9 broth supplemented with Tween 80 and ADC (albumin, dextrose and catalase; Becton Dickinson) for 2 to 3 weeks at 37°C with 100 μg/ml hygromycin for selection, before being reinoculated in fresh broth and used in experiments. Following exposure of 10 mM DETA/NO and SIN-1, clinical strains were also plated in six serial 10-fold dilutions on 7H10 solid media supplemented with OADC (oleic acid, albumin, dextrose and catalase; BD) and incubated at 37°C for 8 weeks.

**Table 1:** Characterization of phenotypic and genotypic antibiotic resistance in *M*. *tuberculosis* H37Rv and clinical strains.

Strain	Resistance pattern (BACTEC 960 MGIT)				Hain result		
	INH	RIF	PZA	EMB	*inhA*	*katG*	*rpoB*
H37Rv	S	S	S	S	WT	WT	WT
H37Rv INH R	R	S	S	S	WT	WT	WT
E3942	S	S	S	S	WT	WT	WT
CDC1551	S	S	S	S	WT	WT	WT
BTB 08–75	R	S	S	S	C-15T	WT	WT
BTB 08–76	R	S	S	S	C-15T	WT	WT
BTB 02–134	R	S	S	S	C-15T	WT	WT
BTB 02–141	R	S	S	S	C-15T	S315T	WT
E1155	R	S	S	S	WT	S315T	WT

### Susceptibility testing of *M*. *tuberculosis* for nitric oxide and peroxynitrite

Phenotypic susceptibility testing of clinical isolates of *M*. *tuberculosis* for INH, rifampicin, pyrazinamide and ethambutol was performed in the BACTEC 460 system (Becton Dickinson) for clinical purposes (Table 1). All clinical strains carrying the pSMT1-plasmid were analysed for common drug resistance mutations to INH and rifampicin using the GenoType MTBDR*plus*® according to the instructions provided by the manufacturer (Hain Lifescience).

For susceptibility testing to NO and peroxynitrite in broth culture, strains were grown as described above, washed in sterile PBS/0.05% Tween 80, adjusted to an optical density (OD) of 0.2–0.8 and diluted to a final concentration of 10^5^ CFU/mL against a standard curve. Diethylenetriamine NONOate (DETA/NO) and ONOO^-^ (3-morpholinosydnonimine, SIN-1 Chloride) (Larodan Fine Chemicals) were diluted (0.1, 1 and 10 mM) in phosphate buffered saline (PBS). Strains were then exposed in Middlebrook 7H9 media supplemented with ADC where DETA/NO or SIN-1 diluted in PBS was added (final PBS concentration 10%). The unexposed control consisted of 10% PBS only and bacteria diluted in Middlebrook 7H9 media supplemented with ADC. A subset of bacterial strains were also exposed to increasing concentrations of PRT (0.032-1mg/L, Sigma-Aldrich) or to INH (2mg/L, Sigma-Aldrich). At baseline (day 0) and after a four day incubation at 37°C, the antimicrobial effect of DETA/NO or SIN-1 in the host-cell free system was determined by measurement of viable bacteria through flash-luminescence with a GloMax® Multi-Detection System (Promega) as previously described [13].

### Susceptibility testing of *M*. *tuberculosis* in a NO-generating macrophage model

Bacterial cultures were washed twice in PBS/0.05% Tween 80 and resuspended in 2ml of DMEM with 10% heat-inactivated FBS (Gibco). The bacterial suspension was added into glass vials containing 2mm glass beads (VWR) and vortexed for 5 min to disrupt aggregates. RAW 264.7 macrophages were infected in triplicates in 96-well plates at a multiplicity of infection (MOI) of 5 and incubated at 37°C in 5% CO_2_. Bacterial uptake, intracellular and extracellular growth was assessed by luminometry after one hour or two days of infection as described previously [13]. To assess the susceptibility of strains to PRT (Sigma-Aldrich), cells were infected as mentioned above in the presence of PRT (0.064 mg/L, 1mg/L and 6 mg/L; Sigma-Aldrich) using 2mg/L of INH (Sigma-Aldrich) as a positive control.

### Determination of nitrite in culture medium

The stable metabolite of NO, nitrite was analysed in cell supernatants with the Griess assay as previously described [14]. In short, the amount of nitrite was assessed by adding sulphanilamide in phosphoric acid immediately followed by N-(1-naphthyl) ethylenediamine (Sigma-Aldrich), and then detected spectrophotometrically at 540 nm followed by transposition to a standard curve.

#### Analysis of resistance mutations and lineage by whole genome sequencing (WGS)

After transformation with the luciferase harbouring pSMT1-plasmid, the strains were sent to the Public Health Agency of Sweden for WGS. Briefly, DNA extracted from cultures growing on Löwenstein-Jensen medium (QIAamp DNA Mini Kit, Qiagen, Hilden, Germany) was sequenced on the Ion Torrent platform (Thermo Fisher Scientific Inc., Waltham, MA, USA). Library preparation and emulsion PCR were performed according to the manufacturer’s instructions. A reference based assembly was performed with CLC Genomics Workbench 8.5.1 (Qiagen, Aarhus, Denmark) using H37Rv (NC000962.3; ATCC 25618) as reference. The sequences reported in this paper have been deposited in European Nucleotide Archive Sequence Read Archive under study number PRJEB199999. Alignment to 4000 alleles of *M*. *tuberculosis* H37Rv (NC_000962.3) was performed and single nucleotide variant (SNV) analysis was done by using SeqSphere+ 3.2.1 (Ridom GmbH, Germany). Only SNVs with a frequency of >75% of substitution-variant to reference-sequence were considered. Known mutations in intergenic sequences such as between *oxyR* and *ahpC* as well as upstream of fabG1 were analysed by manual inspection using the CLC Genomics Workbench. Additionally, known mutations in the following potential bacterial genes associated to NO scavenging products (*ahpC*, *trxA*, *trxB2*, *lpdC*), resistance to PRT (*ddn*) or INH (*katG*, *ahpC or inhA*) were analysed. Lineages were assessed by uploading the fastq-files to the Phylo-Resistance-Search-Engine (PhyResSE, http://phyresse.org) [15].

### Statistics

Samples were run in triplicates and experiments were repeated at least three times. Median values of triplicates were used for calculation of ratios within each experiment and mean values of repeated experiments were used to compare groups. Error bars represent standard error of the mean (SEM). One-way ANOVA with Bonferroni´s multiple comparisons test was used for comparison of multiple groups. Ordinary two-way ANOVA with Tukey´s multiple comparisons test was used for comparison of multiple groups with different stimulations when all means were compared against each other. Ordinary two-way ANOVA with Dunnett´s multiple comparisons test was used for comparisons of multiple groups with different stimulations when means were compared with control mean. In all tests p≤0.05 was considered as significant. All statistical analysis were performed using GraphPad Prism version 6.07.

## Results

### Characterisation of clinical and control strains of *M*. *tuberculosis*

Six clinical strains of *M*. *tuberculosis*, CDC 1551 and the reference strain H37Rv as well as an INH resistant isolate of H37Rv generated by sub-inhibitory concentrations of INH were included in the study (Table 1). The reference strain H37Rv and the clinical strains E3942 Beijing and CDC 1551 were fully susceptible to INH, rifampicin, pyrazinamide and ethambutol by conventional phenotypic drug susceptibility testing (Table 1). In contrast, the remaining clinical isolates were INH resistant, where 08–75, 08–76 and 02–134 had an *inhA* mutation whereas E1155 displayed a *katG* mutation and BTB 02–141 had both *inhA* and *katG* mutations, as determined by the Hain assay and WGS analysis (Tables 1 and 2). The genetic mechanism of H37Rv INH R could not be determined. The growth rate in Middlebrook 7H9 broth did not significantly differ from H37Rv except for BTB 02–134 (S1 Fig). All isolates were from the Beijing or Euro-American linage. The presence of gene variants associated to INH or PRT resistance as well as to factors shown to act as NO scavengers were determined by WGS (Table 2). The susceptibility pattern and analysis of resistance mutations by WGS was not affected by the transformation with the luciferase-harbouring pSMT1-plasmid (Table 2).

**Table 2:** Characterization of lineage and mutations in antibiotic resistance genes (INH and PRT) as well as candidate genes of *M*. *tuberculosis* associated to NO-scavenging effects.

		INH					PRT	NO-scavengers		
Strain	Lineage	*inhA*	*katG*	*ahpC*	*oxyR-ahpC*	*fabG1*	*ddn*	*trxA*	*trxB2*	*lpdC*
H37Rv	Euro-American	WT	WT	WT	WT	WT	WT	WT	WT	WT
H37Rv INH R	Euro-American	WT	WT	WT	WT	WT	WT	WT	WT	WT
E3942	Beijing	WT	WT	WT	WT	WT	WT	WT	WT	WT
CDC 1551	Euro-American	WT	WT	WT	WT	WT	WT	WT	WT	WT
BTB 08–75	Beijing	C-15T	WT	WT	WT	WT	WT	WT	WT	WT
BTB 08–76	Euro-American	C-15T	WT	WT	WT	WT	WT	WT	WT	WT
BTB 02–134	Beijing	C-15T	WT	WT	WT	WT	WT	WT	WT	WT
BTB 02–141	Beijing	C-15T	G315C	WT	WT	WT	WT	WT	WT	WT
E1155	Euro-American	WT	G315C	WT	WT	A747G	WT	C371T	WT	WT

### Exposure to nitric oxide and peroxynitrite induce a strain and dose-dependent growth inhibition of *M*. *tuberculosis*

**Fig 1:**
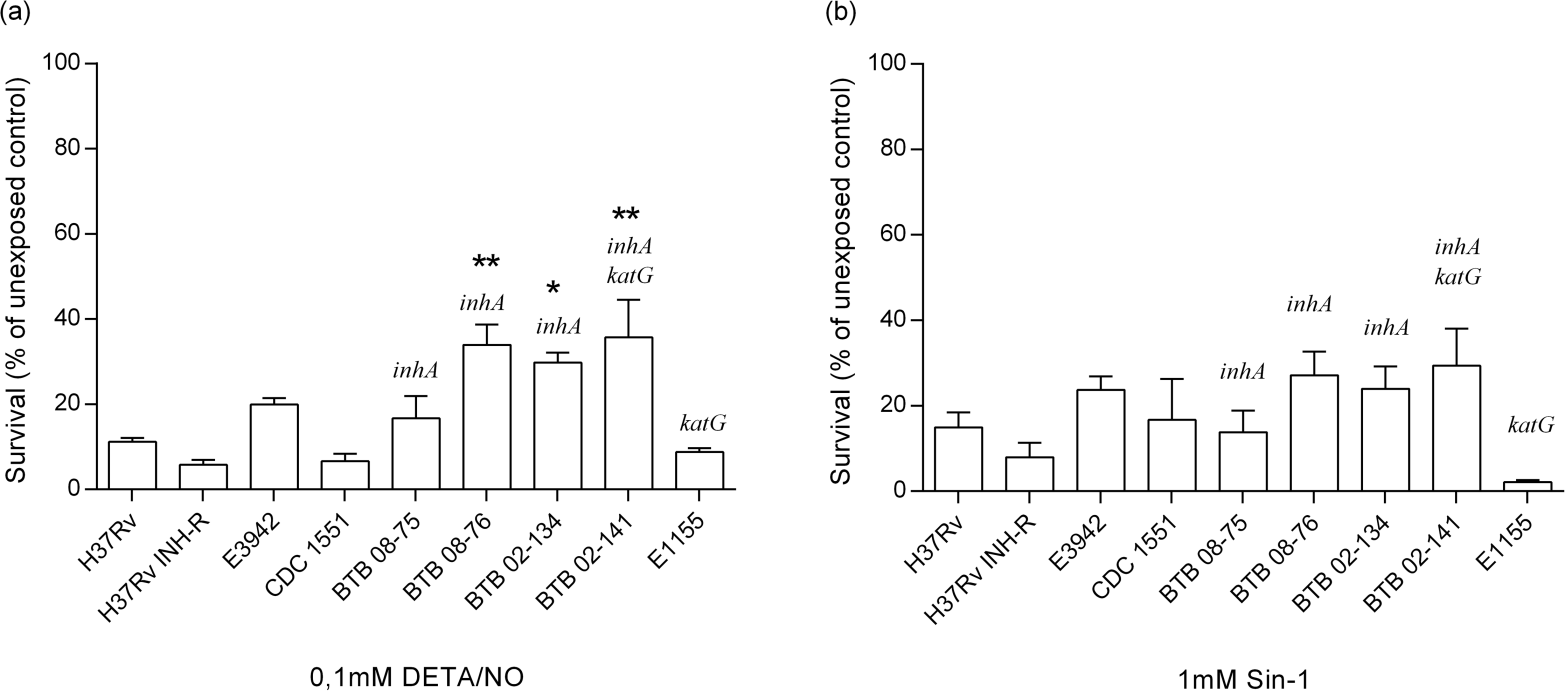
Survival of *M*. *tuberculosis* exposed to DETA/NO and SIN-1. The bacterial strains were exposed to 0.1 mM DETA/NO (a) and 1 mM SIN-1 (b) and bacterial numbers were determined by luminometry after 4 days of incubation. Data are presented as mean bacterial survival (% of unexposed control) ± SEM, (n = 3). Significant differences from H37Rv are indicated with *(p≤0.05), or **(p≤0.01) as determined by one-way ANOVA with correction for multiple testing.

All strains showed a variable, dose-dependent susceptibility to the NO donor DETA/NO and the peroxynitrite donor SIN-1 (S2 Fig). When *M*. *tuberculosis* H37Rv was compared to six clinical isolates, CDC 1551 and the INH resistant H37Rv, three isolates with *inhA* mediated INH resistance (02–141, 02–134 and 08–76) showed significantly increased NO resistance in broth culture (Fig 1). There were no significant differences in the growth of strains at 1mM SIN-1 (Fig 1B) whereas 08–76 exhibited an increased survival at 0.1mM SIN-1 only (S2 Fig). In general, the strains were more susceptible to DETA/NO than to SIN-1 and similar growth inhibition by 0.1mM DETA/NO compared to 10 times higher concentrations of SIN-1 (1mM) was observed in all isolates at 4 days (Fig 1) and at 4h, 24h and 4 days of DETA/NO and SIN-1 exposure for H37Rv and BTB 02–141 (S3 Fig) [16]. At 10 mM of DETA/NO and SIN-1, all strains were killed (S2 Fig), and did not recover after 8 weeks of culturing on 7H10 agar (data not shown).

### Strain variability of intracellular *M*. *tuberculosis* growth in a NO-producing macrophage model

**Fig 2:**
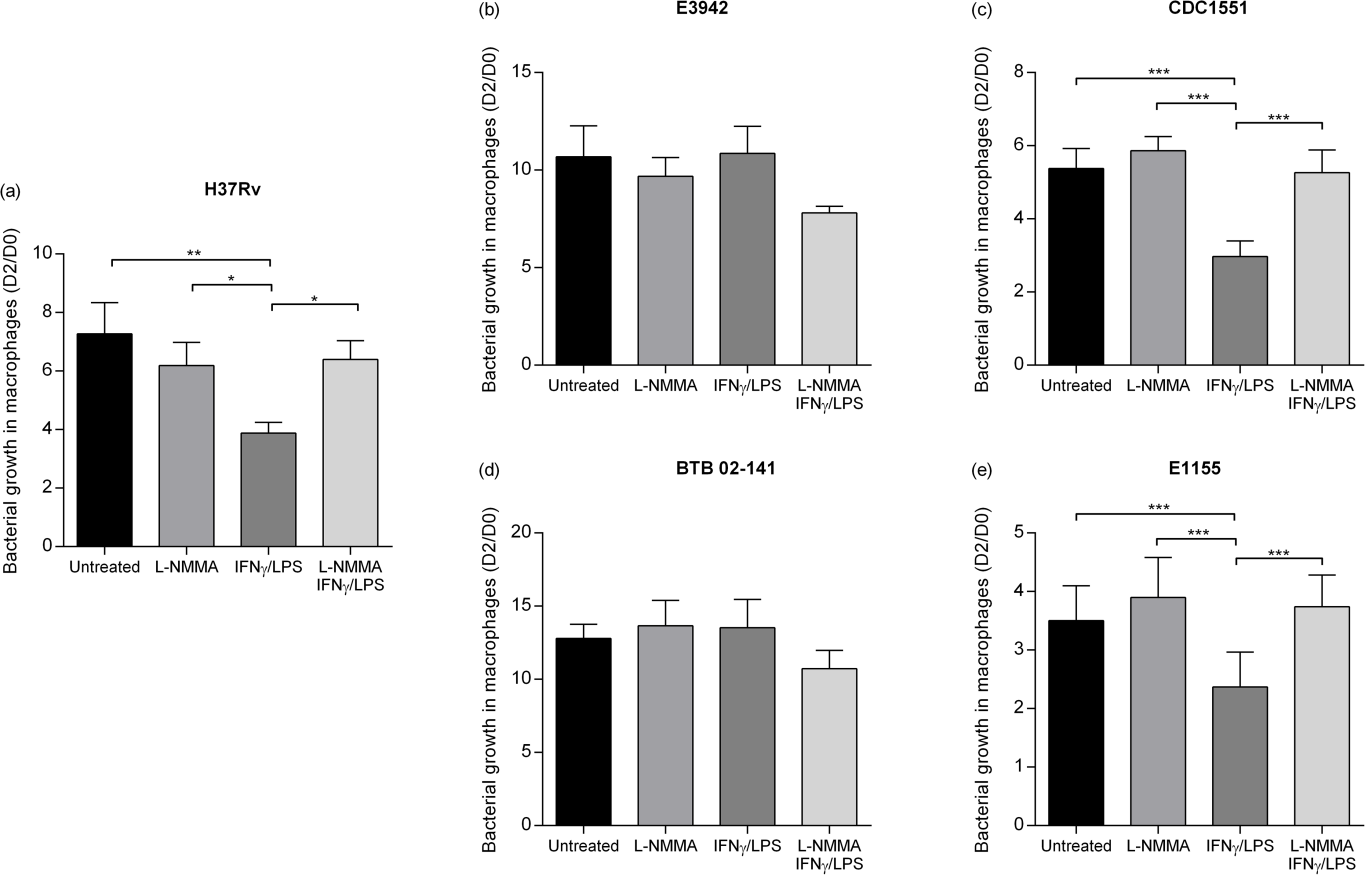
Effect of iNOS inhibition on growth of clinical strains of *M*. *tuberculosis* in macrophages. Macrophages (RAW 264.7) were stimulated with IFN-γ/LPS and/or the iNOS inhibitor L-NMMA (1mM) or left untreated. Macrophages were infected with H37Rv (a), the INH-susceptible strains E3942 (b), CDC 1551 (c), the INH-resistant strains BTB 02–141 (d), and E1155 (e) at a MOI of 5 and bacterial numbers were determined by luminometry. Data are presented as mean fold change of the bacterial numbers on day 2 compared to day 0 (D2/D0) ± SEM, (n = 5–6). Significant differences are indicated with * (p≤0.05), ** (p≤0.01) or with *** (p≤0.001) as determined by one-way ANOVA with repeated measures.

Macrophages (RAW 264.7) were stimulated with IFN-γ/LPS and/or the iNOS inhibitor L-NMMA (1mM) or left untreated before infection with the bacterial strains. None of the combinations significantly altered the phagocytic capacity of the macrophages at one hour post-infection (S4 Fig) and the intracellular fraction showed similar results as the total fraction (Fig 2, S5 Fig). The effective MOI or number of bacteria per macrophage based on the phagocytic capacity after 1h was 1:1 (S4 Fig). Stimulation of macrophages with IFN-γ/LPS significantly reduced the growth of the reference strain H37Rv as well as CDC 1551 and E1155 (Fig 2A, 2C and 2E). This effect was mainly NO-dependent as it was abolished by addition of the iNOS-inhibitor L-NMMA (Fig 2A, 2C and 2E). In contrast, activated macrophages failed to inhibit growth of BTB 02–141 or E3942 and these isolates also showed an increased survival to NO exposure in broth cultures (Figs 1A, 2B and 2D). The levels of the NO metabolite nitrite was significantly increased in IFN-γ/LPS stimulated macrophages compared to IFN-γ/LPS stimulation followed by L-NMMA (S6 Fig).

### Exploratory study on pretomanid susceptibility in the isolate most resistant to NO

**Fig 3:**
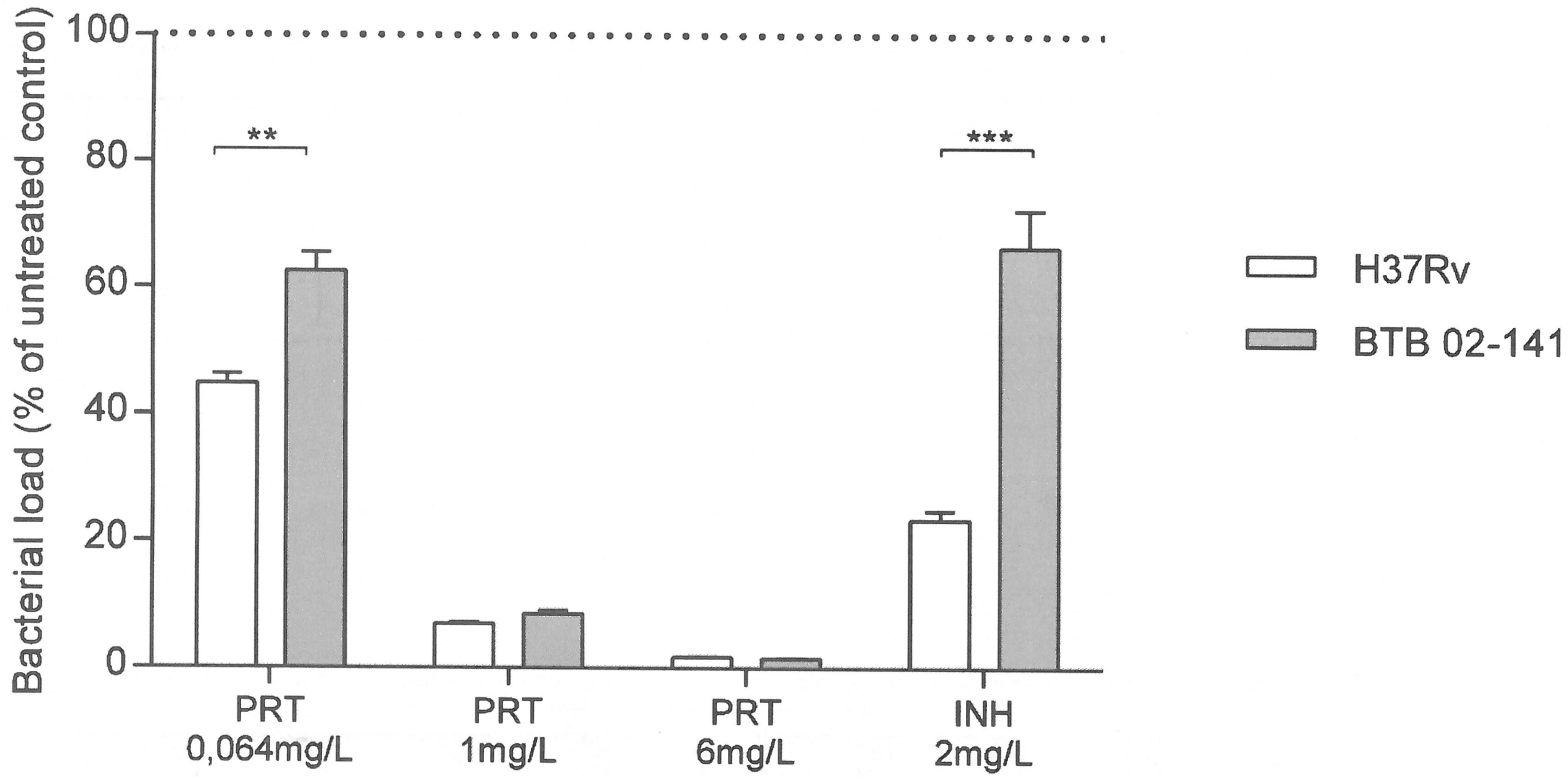
Effect of exposure to isoniazid and pretomanid on intracellular bacterial load. Macrophages (RAW 264.7) were infected with H37Rv or the clinical strain BTB 02–141 at a MOI of 5 and exposed to increasing concentrations of PRT (0.064-2mg/L) or to INH (2mg/L). The bacterial numbers were determined by luminometry at day 2 post infection. Data are presented as mean bacterial survival (% of untreated infected cells) ± SEM, (n = 5–6). Significant differences are indicated with ** (p≤0.01) or *** (p≤0.001) as determined by two-way ANOVA.

**Fig 4:**
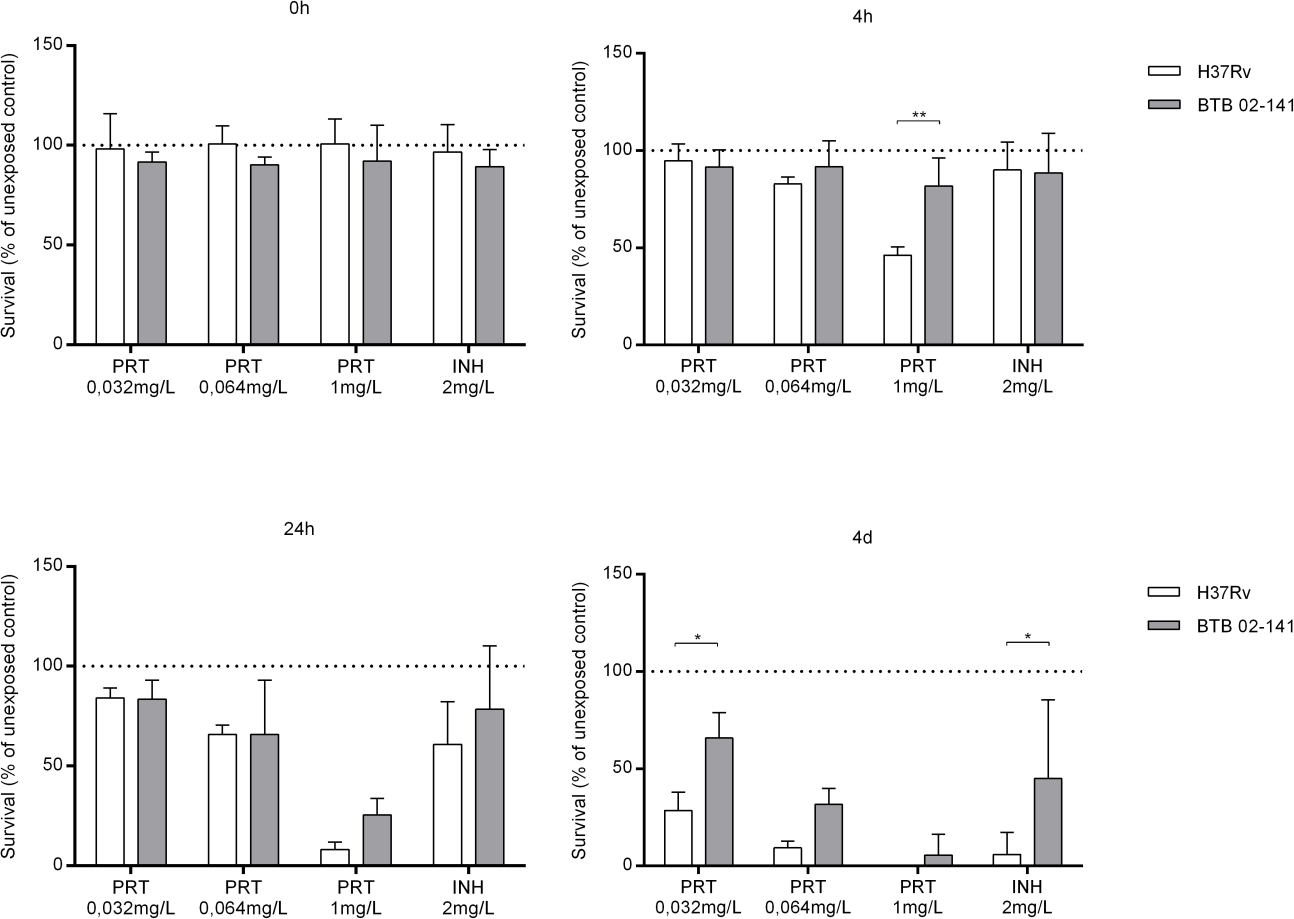
Exposure to pretomanid in broth cultures. The bacterial strains H37Rv and BTB 02–141 were exposed to increasing concentrations of PRT (0.032-1mg/L) or to INH (2mg/L) in broth The bacterial numbers were determined by luminometry directly after exposure (0h) and at 4h, 24h and 4 days later. Data are presented as mean bacterial survival (% of unexposed control) ± SEM (n = 4–5). Significant differences are indicated with * (p≤0.05) or ** (p≤0.01) as determined by two-way ANOVA.

Since activated macrophages did not restrict growth of BTB 02–141 and this isolate also displayed the highest resistance to NO donors in broth culture, we selected this isolate for further intra- and extracellular testing against the NO-inducing drug PRT. The reference strain H37Rv was included for comparison. There was a dose-dependent reduction in intracellular bacterial growth of both the reference strain H37Rv and the INH-resistant clinical strain BTB 02–141 when RAW 264.7 macrophages were exposed to increasing concentrations of PRT (Fig 3). The INH-resistant clinical strain BTB 02–141 was however significantly more resistant to low concentrations (0.064 mg/L) of PRT compared to the reference strain H37Rv. There was a similar pattern when the isolates were exposed to PRT in broth where BTB 02–141 was significantly more resistant at 0.032 mg/L PRT compared to H37Rv (Fig 4).

## Discussion

In this study, we investigated the effect of reactive nitrogen species on clinical strains of *M*. *tuberculosis*, both in broth cultures and in an NO-generating macrophage model. Our results in a limited number of strains show a variability in the susceptibility to NO which may have an impact on anti-mycobacterial drugs acting through NO such as PRT.

In support of our findings, *M*. *tuberculosis* strains showed a similar pattern of NO-dependent growth suppression both in broth cultures and in NO-producing macrophages. Other factors known to restrict growth of intracellular *M*. *tuberculosis* include the antimicrobial peptide cathelicidin and reactive oxygen radicals [17]. However, the inclusion of the NO-inhibitor L-NMMA showed that growth suppression in IFNγ- and LPS-activated macrophages was mainly NO-dependent. This observation is in line with previous studies using J774 and RAW cells [18, 19]. Local concentrations of NO in the cell-associated environment is expected to be in the range of 40μM to 1mM [20], which is comparable to the concentrations generated in our experiments with DETA/NO.

In a limited number of studies, INH-resistant strains have been shown to be more tolerant to NO [21, 22]. The correlation between NO resistance and resistance mutations in *katG* only was less clear from our findings, but interestingly, all isolates showing increased NO resistance in broth cultures had mutations in *inhA* including the BTB 02–141 strain exhibiting mutations both in *inhA* and *katG*. The molecular mechanisms of INH resistance is complex, and can only partly be explained by resistance mutations in *inhA*, *katG* and *ahpC* [23]. Indeed, the molecular mechanism of resistance could not be established for the in vitro selected H37Rv INH R isolate. The *katG* gene encodes for the enzyme catalase peroxidase, a scavenger for ROI and RNS [24], but is also needed to activate INH [25]. Clearly, there is a need of further studies with larger sample sizes in order to adequately address the association between INH resistance and reduced susceptibility to NO.

In the intracellular, NO generating macrophage model we found that the BTB 02–141 strain was not inhibited which correlated to the significantly increased survival following NO exposure in broth. For the E1155 isolate with a *katG* mutation, there was a significant growth control in activated macrophages which was associated to a higher NO susceptibility in broth cultures compared to H37Rv. Although more data is clearly needed, this indicates that NO resistance needs to be investigated not only in relation to phenotypic INH resistance but also according to the mechanism of INH resistance. Activated macrophages did not restrict growth of the INH susceptible E3942 isolate, and the same isolate showed a non-significant but reduced susceptibility in broth compared to H37Rv. The results from the limited number of clinical isolates tested in the intracellular model indicates that there may be several mechanisms unrelated to INH resistance associated to a failure of activated macrophages to control isolates with reduced NO susceptibility in broth culture.

Bacteria that survive the exposure to NO may reflect selected populations of *M*. *tuberculosis* adapted to resist NO. Such defence mechanisms are poorly defined but include upregulation of ahpC encoding alkyl hydroperoxide reductase subunit C [21, 26], lpd encoding lipoamide dehydrogenase [27], the thioredoxin systems [28], and the dormancy survival regulator DosR [29]. In an effort to identify potential mutations in the genes involved in the bacterial defence against NO, we sequenced all the isolates, but no explanation for the increased NO-tolerance in BTB 02–141 could be found.

Yu et al [16] used a different experimental approach from ours with direct exposure to ONOO^-^ and NO. It was shown that virulent *M*. *tuberculosis* strains were more susceptible to NO than to ONOO^-^ compared to BCG and *M*. *smegmatis*, which is in line with our data showing that ten times higher concentrations of SIN-1 were needed to show similar inhibition of isolates compared to DETA/NO including earlier time points of exposure (4h and 24h). However, differences in the half-life and chemical composition of DETA/NO and SIN-1 may influence the comparison between NO and ONOO^-^ susceptibility and the results should be interpreted with caution. Previous data have indicated that the occurrence of NO-resistant strains of *M*. *tuberculosis* [30, 31], such as CDC 1551, is responsible for human outbreaks of *M*. *tuberculosis* [32]. However, our results show a susceptibility to RNS for CDC 1551 at the same level as *M*. *tuberculosis* H37Rv. One reason for the observed differences may be that in previous assays for determining NO-tolerance, acidified sodium nitrite was used [32]. Such studies have been limited by the use of strategies for NO generation by NaNO_2_ requiring a low pH, that *per se* [33] can affect the growth of *M*. *tuberculosis*. This is not the case to the same extent for NO-donors such as DETA/NO (23). Furthermore it is difficult to control which RNS is produced from acidified sodium nitrite in liquid solutions at low pH [34]. DETA/NO is a small molecule, which has been commonly used in assays of NO exposure [35]. The half-life of DETA/NO and SIN-1 is 20 hours and 15–30 minutes respectively and there are very limited effects of the donating agents themselves [36, 37].

We found that activated macrophages could not restrict growth of the most NO-resistant isolate in broth cultures (BTB 02–141). Interestingly, BTB 02–141 was also more resistant to PRT in the macrophage model compared to H37Rv and this was also the case upon exposure to PRT in broth cultures. Previous data show that the main anaerobic effect of PRT is mediated by the release of NO whereas the aerobic effect is believed to be mainly a result of disrupting the formation of mycolic acids which are important in the mycobacterial cell wall [7, 8]. However, NO is also generated from PRT exposure under aerobic conditions and the relative contribution of bacterial NO resistance under aerobic and anaerobic conditions remains to be elucidated. We tested PRT concentrations in the range of 0.032 mg/L to 1 mg/L as the MIC distribution has been shown to be in that range in broth culture [38]. Previous data indicate a C_max_ of 1.2 mg/L in plasma from a 200mg dose of PRT in adults which has been used in clinical trials [39]. Thus, C_max_ levels are 20–40 times higher than the MICs where we found significant differences in bacterial survival between H37Rv and the clinical isolate 02–141 both in broth cultures (Fig 4; 0.032 mg/L) and the intracellular model (Fig 3; 0.064 mg/L). Our limited and preliminary observations regarding to co-existence of NO resistance and reduced susceptibility to PRT in one clinical isolate relative to H37Rv should be interpreted with caution but further explored as it may have therapeutical implications for the use of nitroimidazoles.

Our study has several limitations and the major one is the limited number of strains included. In particular, the association between PRT and NO resistance is reported for only one clinical isolate relative to H37Rv and needs to be confirmed in a representative selection of clinical isolates. It should be emphasized that the exact mechanisms involved in scavenging of RNS in *M*. *tuberculosis* remains poorly defined and the mechanisms for variability in NO susceptibility among clinical isolates should be further explored. Mycobacterial defence mechanisms to NO may be targets for new drug candidates. Of note, the mechanisms of NO susceptibility may also be different in the intracellular environment which is illustrated by recent data showing that NO stress in murine macrophages induce drug tolerance in *M*. *tuberculosis* [40].

In conclusion, our results show a variability in NO susceptibility among clinical isolates of *M*. *tuberculosis*. Mycobacterial NO resistance may have an impact on the treatment of tuberculosis as key drugs such as INH and PRT partly mediate their anti-mycobacterial effects through NO. Further studies are needed to substantiate the underlying mechanisms as well as the clinical relevance of these findings for the treatment of active and latent tuberculosis.

## Supporting information

S1 Fig Growth in brothof different strains of *M*. *tuberculosis*. The strains were cultured in Middlebrook 7H9 medium supplemented with Tween 80 and ADC and bacterial load was determined by luminometry. Data are presented as mean log_10_ of fold change in bacterial numbers between day 4 and day 0 (log 10 of D4/D0), (n≥3). Significant differences from H37Rv is indicated with *(p ≤ 0.05) as determined by one-way ANOVA.
(TIF)

S2 Fig Dose dependent effects of DETA/NO and SIN-1 against M. tuberculosis. The bacterial strains were exposed to increasing concentrations of DETA/NO (a) and SIN-1 (b) and bacterial numbers weres determined by luminometry. Data are presented as mean bacterial survival (% of unexposed control) after 4 days of incubation ± SEM, (n = 3). Significant differences from H37Rv are indicated with * (p ≤ 0.05), or *** (p≤0.001) as determined by two-way ANOVA.
(TIF)

S3 Fig Bacterial load in broth cultures exposed to DETA/NO and 1mM SIN-1. The H37Rv and BTB 02–141 were exposed to 0.1mM DETA/NO (a) and 1mM SIN-1 (b). Bacterial load after 4hours, 24h and 4 days later was determined by luminometry. Data are presented as mean bacterial survival (% of unexposed control) ± SEM, (n = 4–5). Significant differences from H37Rv are indicated with * (p ≤ 0.05), or ***(p≤0.001) as determined by two-way ANOVA.
(TIF)

S4 Fig Phagocytosis of clinical strains. Macrophages (RAW 264.7) were stimulated with IFN-γ/LPS and/or iNOS inhibitor L-NMMA or left untreated. Phagocytosis of the reference strain H37Rv (a), the INH-susceptible strains E3942/94 Beijing (b), CDC 1551 (c) the two INH-resistant strains BTB 02–141 (d) and E1155/94 (e) by macrophages was measured by determining bacterial load using luminometry after one hour of infection at MOI of 5. Data are presented as a mean ratio of intracellular bacteria divided by the sum of intracellular and extracellular bacteria ± SEM (n = 5–6). Differences between groups were analysed by one-way ANOVA with repeated measures.
(TIF)

S5 Fig Intracellular survival of M tuberculosis clinical isolates. Macrophages (RAW 264.7) were stimulated with IFN-γ/LPS and/or the iNOS inhibitor L-NMMA (1mM) or left untreated. Macrophages were infected with H37Rv (a), the INH-susceptible strains E3942 (b), CDC 1551 (c), the INH-resistant strains BTB 02–141 (d), and E1155 (e) at a MOI of 5. Bacterial load was measured at day 0 and day 2 using luminometry. Data are presented as mean fold change of the intracellular bacterial numbers on day 2 divided by day 0 (D2/D0) ± SEM, (n = 5–6). Significant differences are indicated with * (p≤0.05) or ** (p≤0.01) as determined by one-way ANOVA with repeated measures.
(TIF)

S6 Fig The nitrite level in supernatants of activated macrophages after treatment with L-NMMA. Macrophages (RAW 264.7) were stimulated with IFN-γ/LPS alone or in combination with 1mM iNOS inhibitor L-NMMA. Nitrite levels were measured with the Griess assay. The lowest concentration within the linear range of the standard curve was considered as detection limit of the assay and. All values lower than that were assigned below detection limit (BDL). Data are presented as mean of nitrite concentration ± SEM, (n = 5).
(TIF)
