# Supplementary figures and images for: Reduced susceptibility of clinical strains of Mycobacterium tuberculosis to reactive nitrogen species promotes survival in activated macrophages
Source: PLoS One. 2017 Jul 13;12(7):e0181221. doi: 10.1371/journal.pone.0181221 (PMC5509328; doi:10.1371/journal.pone.0181221)

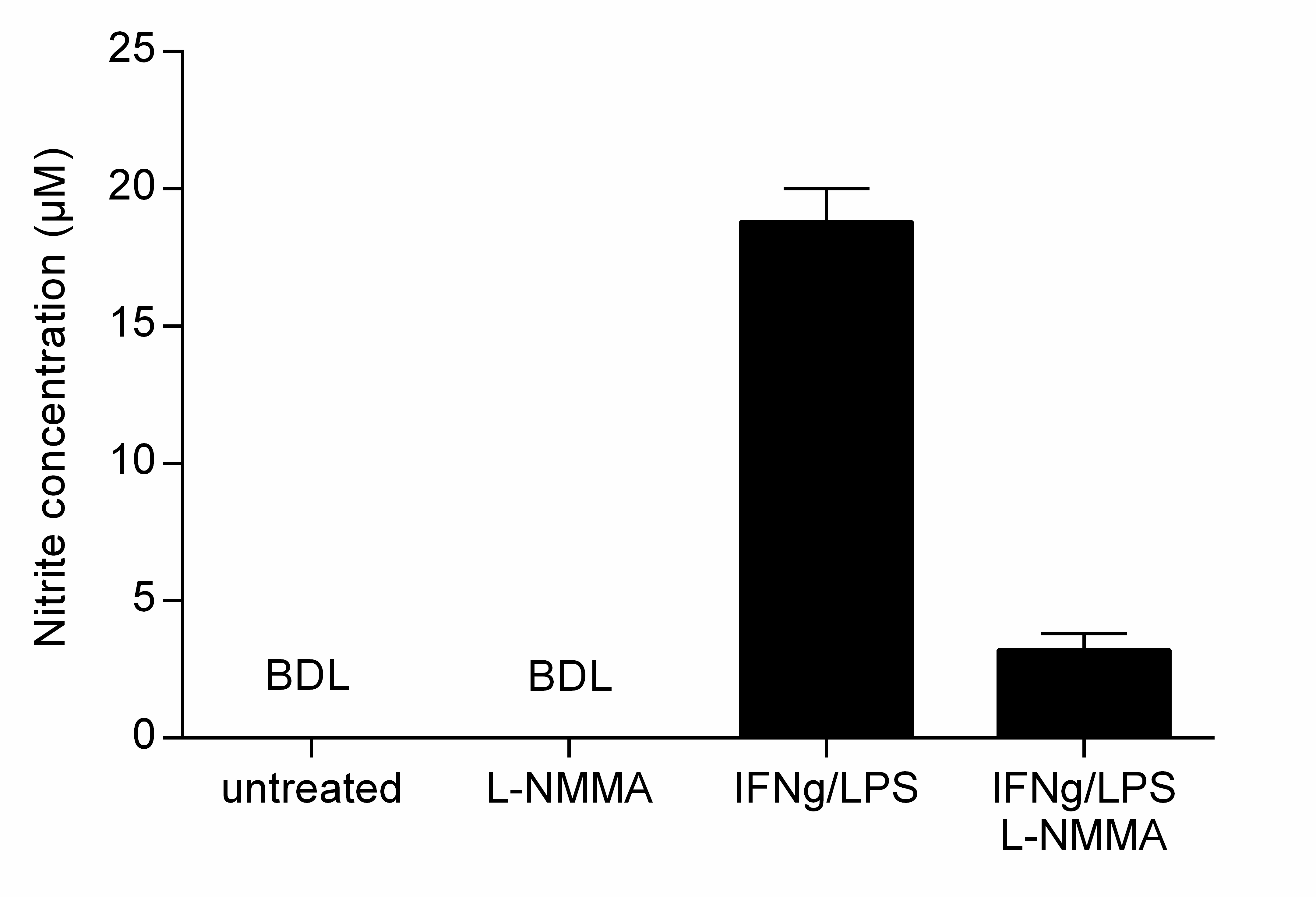

Supplement: S6 Fig — Macrophages (RAW 264.7) were stimulated with IFN-γ/LPS alone or in combination with 1mM iNOS inhibitor L-NMMA. Nitrite levels were measured with the Griess assay. The lowest concentration within the linear range of the standard curve was considered as detection limit of the assay and. All values lower than that were assigned below detection limit (BDL). Data are presented as mean of nitrite concentration ± SEM, (n = 5). (TIF) [file pone.0181221.s006.tif]
